# Supplementary material for: Novel Evolutionary Engineering Approach to Alter Substrate Specificity of Disaccharide Transporter Mal11 in Saccharomyces cerevisiae
Source: J Fungi (Basel). 2022 Mar 30;8(4):358. doi: 10.3390/jof8040358 (PMC9024999; doi:10.3390/jof8040358)
Supplement: Supplementary file 1 [file jof-08-00358-s001.zip › Figure S1.pdf]

Supplementary materials

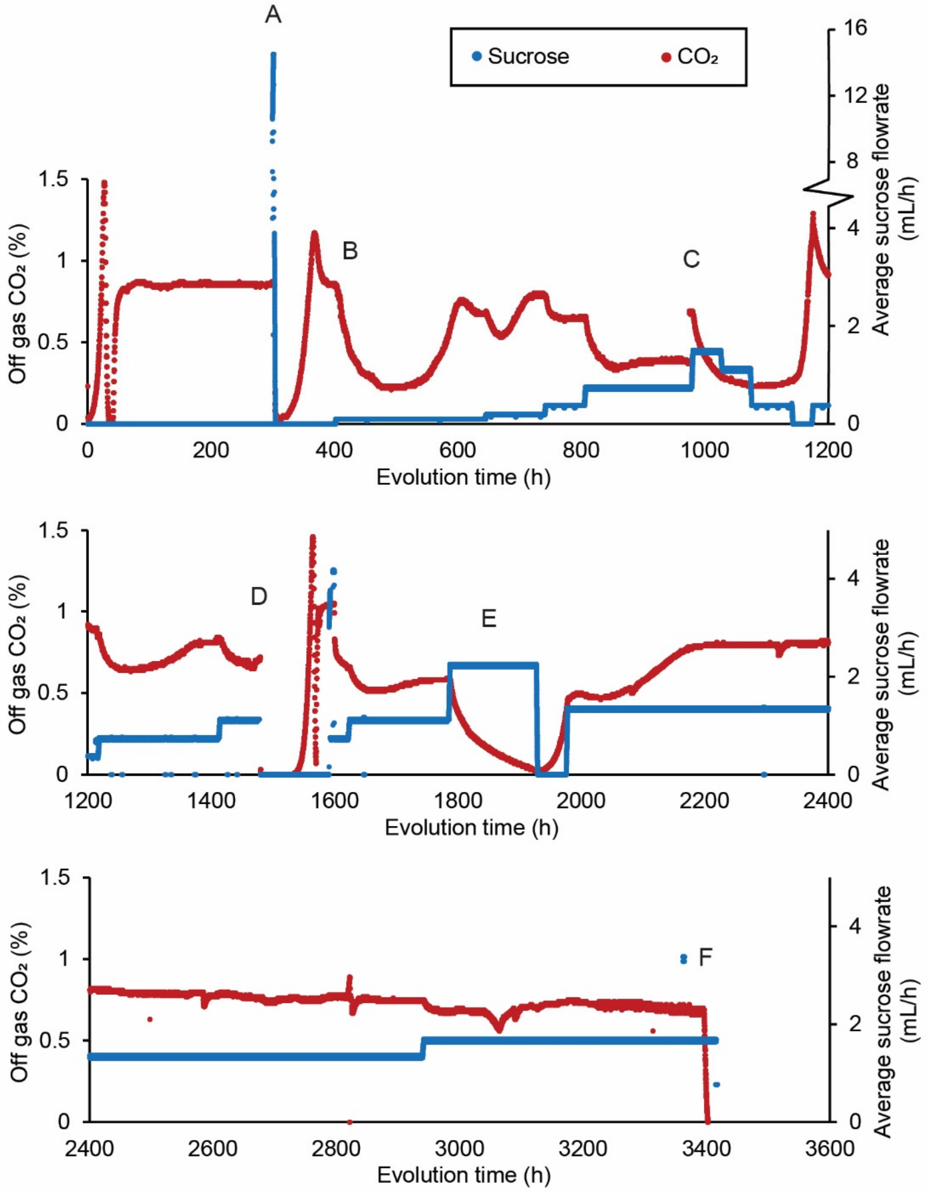

Figure S1: CO_2 concentrations in the reactor off gas and average flowrate of a 500 g L^{-1} sucrose solution during adaptive laboratory evolution of IMZ786 (*sugar⁰ MAL11*) in anaerobic glucose-limited chemostats with pulse-wise addition of sucrose. The off gas CO_2 concentrations were used as a read-out to monitor growth. One representative of the two independent evolution experiments is shown. **A**: After a stable CO_2 concentration of ~0.83% in the reactor off gas was observed, sucrose was administered separately to the culture at an average flowrate of 16 mL h^{-1}. At this average flowrate, the CO_2 concentration decreased to zero. To subsequently prevent washout of cells

and allow for evolution, the addition of sucrose was temporarily stopped. **B**: After ~400 h, the average sucrose flowrate was set to 0.09 mL h^{-1}. This resulted in a decrease of the off-gas CO_2 concentration to a value of 0.22%. **C**: After 975 hours, there was a temporal disturbance (increase) in the constant medium flowrate as a new medium vessel was connected, resulting in a sudden peak in off gas CO_2 concentration (0.63%, whereas before connection of the medium vessel it was relatively stable at ~0.37%). After this peak, the ever-decreasing CO_2 concentration motivated the decision to decrease the average sucrose flowrate stepwise, until an increase of the CO_2 concentration was once again observed as no more sucrose was added. **D**: Due to laboratory maintenance, the cultures were temporarily stocked and terminated. After approximately one month, these stocks were used to proceed with the evolution. **E**: The average sucrose flowrate (2.2 mL h^{-1}) set after ~1785 h resulted in gradual wash-out of cells, as can be observed from the decreasing off gas CO_2 concentration (from 0.58% to 0.02%). Addition of sucrose was therefore temporarily stopped to allow for accumulation of biomass, after which the average flowrate of the sucrose solution was adjusted to a lower setting that still permitted sufficient growth. **F**: After ~3400 h, aliquots of the cultures were stocked and the evolution experiment was terminated.

Separate excel files for Supplementary Data S1 and Supplementary Data S2 have been included.
